# Supplementary material for: A Comparative Study of the Adherent-Invasive Escherichia coli Population and Gut Microbiota of Healthy Vegans versus Omnivores
Source: Microorganisms. 2020 Jul 31;8(8):1165. doi: 10.3390/microorganisms8081165 (PMC7465959; doi:10.3390/microorganisms8081165)
Supplement: Supplementary file 1 [file microorganisms-08-01165-s001.pdf]

Supplementary Table 1. Primers used in this study.

Gene	Primer code	Primer Sequence '5 → 3'	Amplicon size (bp)	Ref.
*afa*C	afaC F<br>afaC R	CGGCTTTTCTGCTGAACTGGCAGGC<br>CCGTCAGCCCCCACGGCAGACC	627	(4)
*lpf*A	lpfA F<br>lpfA R	AGGCGGTGCATTCACTCTGGCATCT<br>CCGCGTCGATAGCGGTATAGGCAGA	448	(4)
*htr*A	htrA F<br>htrA R	TTCCAGCAGTTCTTCGGTGA<br>ATCAGTTCGCCGTTCAGGTT	530	(4)
*dsb*A	dsbA F<br>dsbA R	CTGCCGGAAGGCGTGAAC<br>GCTGTTCCACGCCGCGTC	237	(4)
*clb*A	clbA F<br>clbA R	CTAGATTATCCGTGGCGATTC<br>CAGATACACAGATACCATTCA	872	(4)
*omp*C	ompC F<br>ompC R	GCGCCGACATCAACGTATTT<br>GCCAACAAAGCGCAGAACTT	141	(4)
*usp*A	uspA F<br>uspA R	CCGATACGCTGCCAATCAGT<br>ACGCACACCGTAGGCCAGAT	288	(27)

Supplementary Table 2. Polymerase chain reaction (PCR) cycling conditions for amplification of six adherent-invasive *Escherichia coli* (AIEC)-associated virulence genes (VGs) and *usp*A gene.

Test	Initial Denaturation	Time (min)	Denaturation Temp. (°C)	Time	Annealing Temp. (°C)	Time	Extension Temp. (°C)	Time (min)	No. of Cycles	Final Extension	Time (min)
***afa*C**	94°C	2	94°C	1	65°C	1	72°C	2	30	72°C	5
***lpf*A**	95°C	2	95°C	1	65°C	1	72°C	1	30	72°C	5
***htr*A**	95°C	2	95°C	1	55°C	1	72°C	2	30	72°C	5
***dsb*A**	95°C	2	95°C	30 s	56°C	1	72°C	2	30	72°C	5
***clb*A**	95°C	2	95°C	30 s	56°C	1	72°C	2	30	72°C	5
***omp*C**	95°C	2	95°C	30 s	53°C	1	72°C	2	30	72°C	5
*usp*A	94°C	5	94°C	2	70°C	1	72°C	1	30	-	-

Supplementary Table 3. Presence of AIEC-associated VGs among vegans and omnivores. CT 1–CT 59 represent vegans, and CT 60–CT 99 represent omnivores. The highlighted sections indicate the CTs analyzed in triplicate, which were used as a control to demonstrate the true representation of the selected isolates for further analysis.

CT	*htr*A	*lpf*A	*omp*C	*clb*A	*dsb*A	*afa*C	No. of *E. coli* Represented
CT 1	+		+		+	+	9
CT 2	+		+				2
CT 3	+		+				4
CT 4	+		+				2
CT 5	+	+	+		+		5
CT 6	+	+	+		+		27
CT 7	+	+	+		+		27
CT 8	+	+	+	+	+	+	8
CT 9	+		+	+	+		2
CT 10			+				3
CT 11	+	+	+		+	+	7
CT 12-1	+		+	+	+		28
CT 12-2	+		+	+	+		
CT 12-3	+		+	+	+		
CT 13	+		+	+	+		28
CT 14	+		+		+		6
CT 15	+		+		+		2
CT 16	+		+		+		2
CT 17	+		+		+		16
CT 18	+		+		+		28
CT 19	+		+		+		14
CT 20	+	+	+		+		2
CT 21	+		+				4
CT 22	+		+				4
CT 23	+	+	+		+	+	28
CT 24	+		+				28
CT 25	+		+	+	+		7
CT 26	+		+		+	+	6
CT 27	+		+		+	+	8
CT 28	+		+		+		6
CT 29	+	+	+		+		27
CT 30	+		+		+	+	18
CT 31	+	+	+	+		+	10
CT 32	+	+	+	+			28
CT 33	+	+	+	+			28
CT 34	+	+	+	+		+	28
CT 35	+	+	+	+		+	28
CT 36	+		+	+			5
CT 37	+		+	+			2
CT 38			+				15
CT 39			+				3
CT 40	+		+	+		+	28
CT 41	+	+	+	+		+	28
CT 42	+		+	+		+	28
CT 43	+	+	+	+		+	25
CT 44	+	+	+	+			3
CT 45	+		+	+			24
CT 46	+		+	+			4
CT 47	+	+	+	+			15

CT 48	+	+	+	+			10
CT 49	+	+	+	+			3
CT 50	+	+	+	+			28
CT 51	+	+	+		+		28
CT 52	+	+	+		+	+	26
CT 53	+		+		+		28
CT 54	+	+	+		+	+	27
CT 55	+	+	+		+	+	26
CT 56	+		+	+			2
CT 57	+		+		+		28
CT 58	+	+	+		+		28
CT 59	+		+		+		28
CT 60	+		+	+	+		28
CT 61	+	+	+		+	+	9
CT 62	+	+	+		+	+	6
CT 63	+	+	+		+	+	10
CT 64	+	+	+		+	+	2
CT 65	+		+	+	+		3
CT 66	+	+	+		+	+	23
CT 67	+		+	+	+		22
CT 68	+	+	+		+	+	6
CT 69-1	+	+	+		+	+	
CT 69-2	+	+	+		+	+	28
CT 69-3	+	+	+		+	+	
CT 70-1	+		+	+	+		
CT 70-2	+		+	+	+		24
CT 70-3	+		+	+	+		
CT 71	+	+	+			+	4
CT 72	+	+	+		+	+	25
CT 73	+		+	+	+		28
CT 74	+		+				28
CT 75	+		+		+		28
CT 76	+		+				28
CT 77	+		+		+		2
CT 78	+	+	+		+		26
CT 79	+	+	+		+	+	28
CT 80	+		+	+		+	15
CT 81	+	+	+	+		+	13
CT 82	+	+	+	+		+	27
CT 83	+	+	+	+		+	27
CT 84	+		+	+			16
CT 85	+		+	+			2
CT 86	+	+	+	+			4
CT 87	+		+		+		28
CT 88	+		+		+	+	27
CT 89	+		+		+		28
CT 90	+	+	+		+		28
CT 91	+		+		+		28
CT 92	+		+	+	+		28
CT 93	+		+		+	+	28
CT 94	+	+	+		+		28
CT 95	+	+	+		+		17
CT 96	+		+		+	+	4
CT 97	+	+	+		+	+	5
CT 98	+		+				24
CT 99	+		+				3

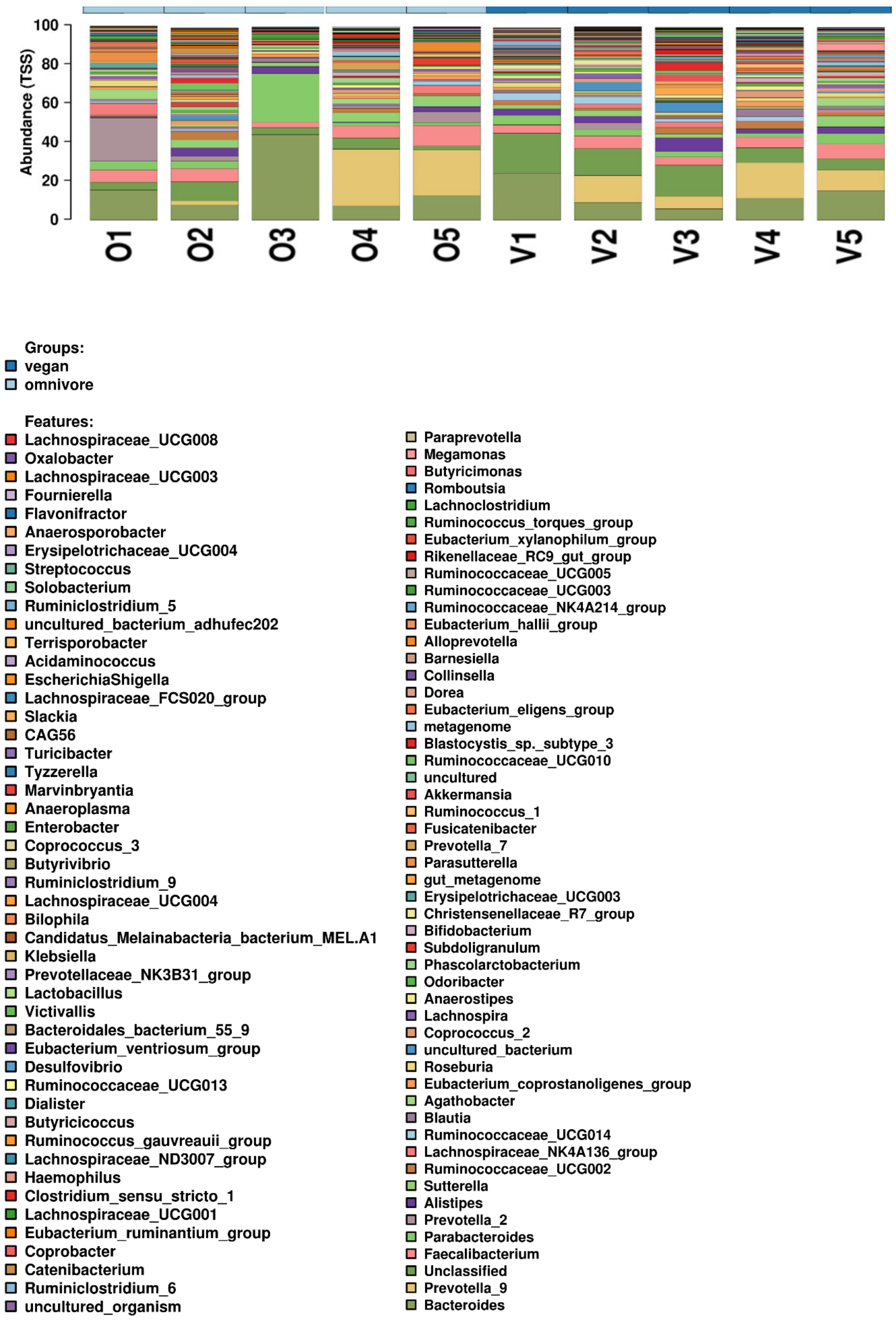

Supplementary Figure 1. Genus level diversity analysis of the top 100 genera using 5000 OTUs in vegan and omnivore composite samples. Composite samples were composed of one to four samples. O1-O5 represent omnivores, and V1-V5 represent vegans. TSS: total sum scaling, i.e., normalized proportional abundance.
